# Supplementary figures and images for: Optimisation strategies for directed evolution without sequencing
Source: PLoS Comput Biol. 2024 Dec 19;20(12):e1012695. doi: 10.1371/journal.pcbi.1012695 (PMC11698521; doi:10.1371/journal.pcbi.1012695)

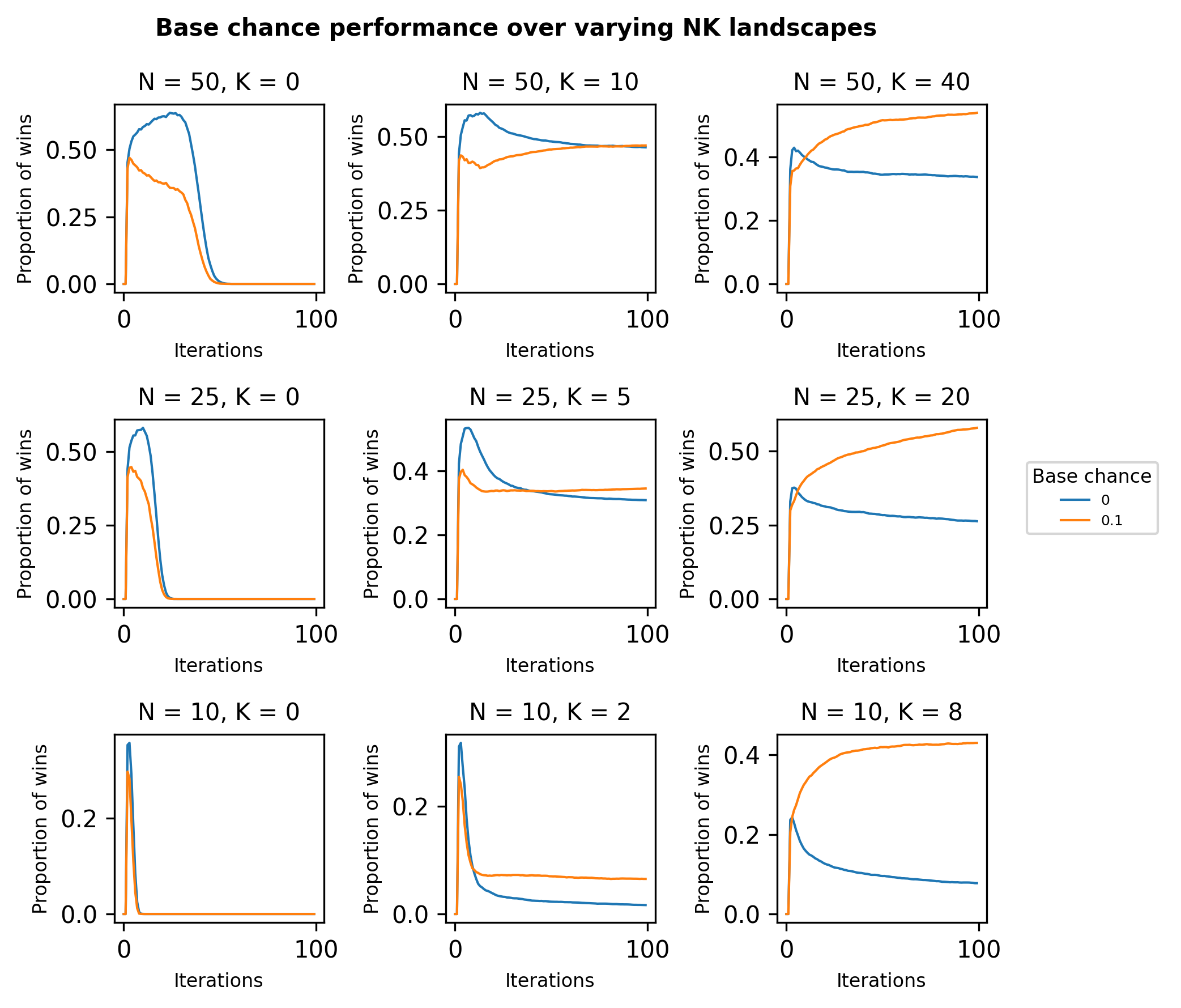

Supplement: S1 Fig — Data shows the proportion of runs that are won by either a 0 base chance, or 0.1 base chance strategy over different landscapes. Where the outcome is tied, the value is 0. Value of N increases vertically, and the value of K/N increases up horizontally. Mutations per cell = 0.1, population size = 1000. (TIF) [file pcbi.1012695.s001.tif]

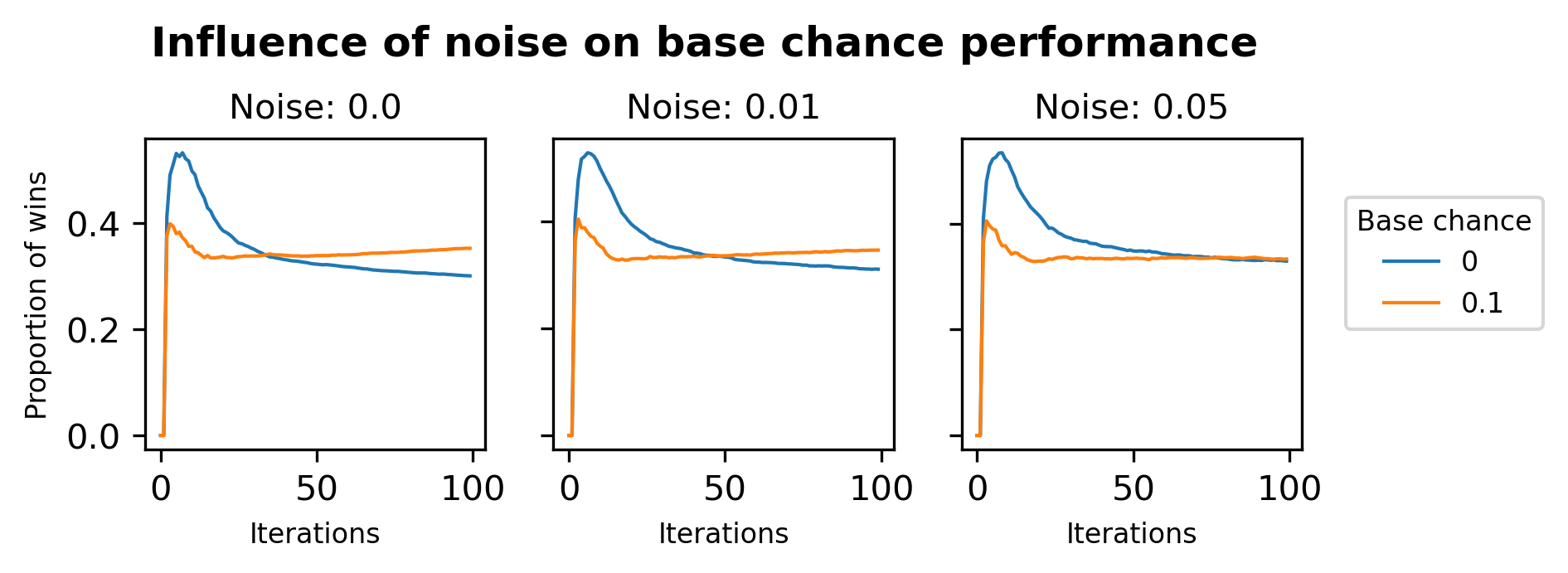

Supplement: S2 Fig — To introduce noise, values sampled from N(0,noise2) are added to fitness values each generation. N = 25, K = 5, mutations per cell = 0.1, population size = 1000. (TIF) [file pcbi.1012695.s002.tif]

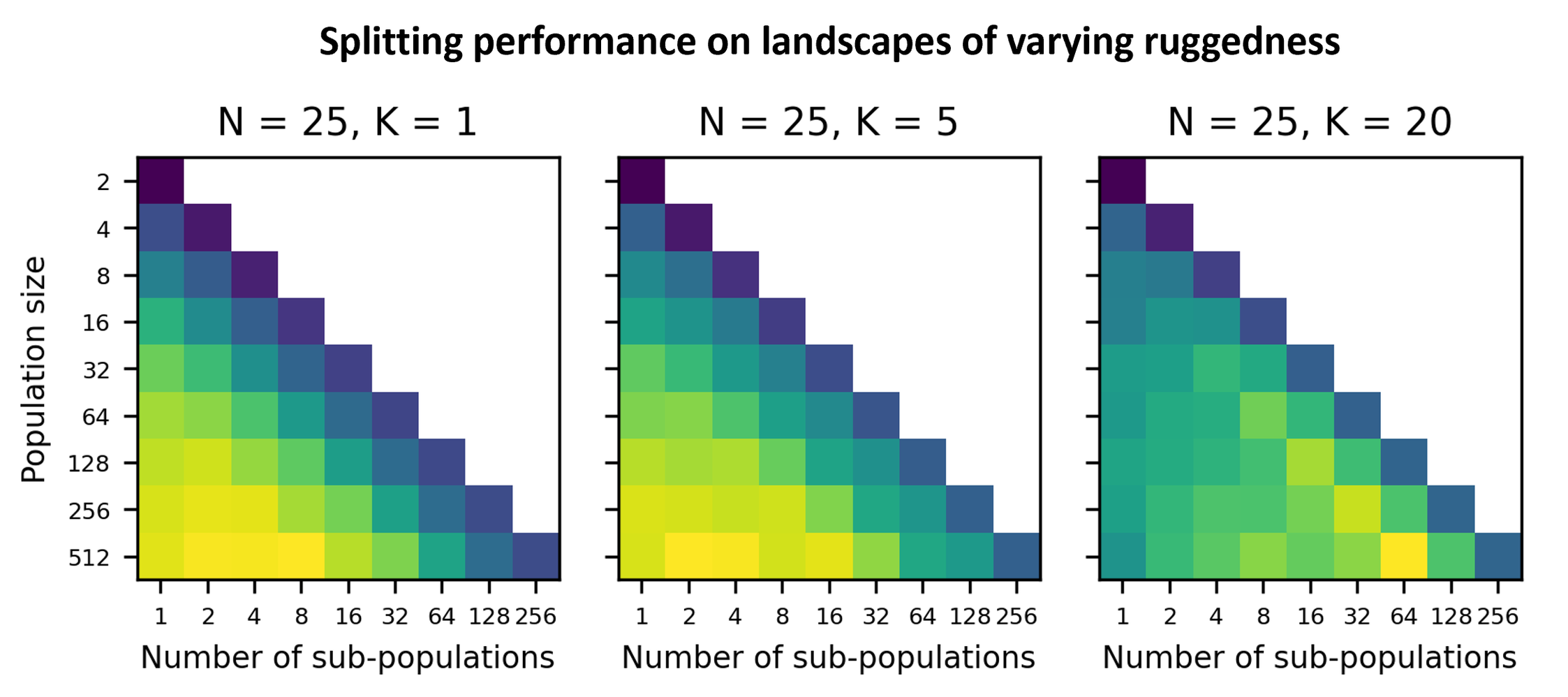

Supplement: S3 Fig — Mutations per cell = 0.1. (TIF) [file pcbi.1012695.s003.tif]

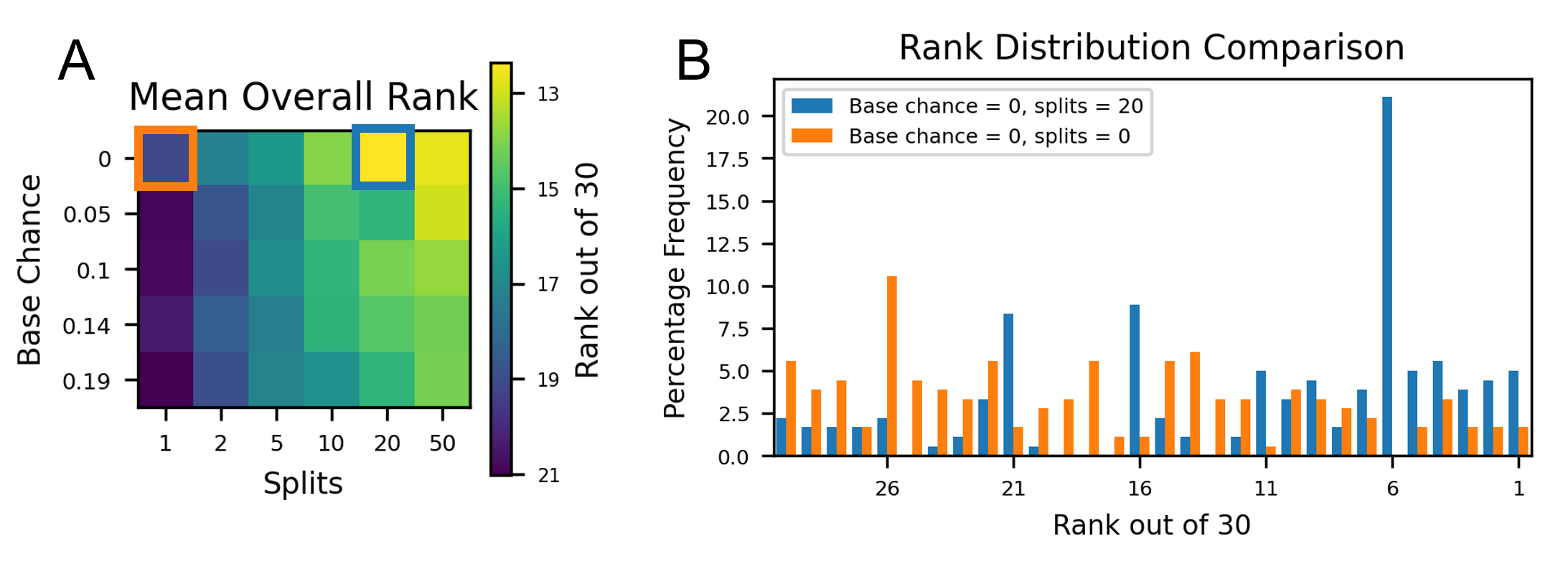

Supplement: S4 Fig — Results were processed by ranking each of the 30 strategies (combinations of base chance and splitting) against one another for each set of parameters. A: Average rank overall. B: Histogram of ranks, top performing strategy vs standard strategy (no base chance, no splits). (TIF) [file pcbi.1012695.s004.tif]
