# Supplementary material for: Optimisation strategies for directed evolution without sequencing
Source: PLoS Comput Biol. 2024 Dec 19;20(12):e1012695. doi: 10.1371/journal.pcbi.1012695 (PMC11698521; doi:10.1371/journal.pcbi.1012695)
Supplement: Attachment — Submitted filename: Comment_Response_Letter.pdf [file pcbi.1012695.s006.pdf]

To the Editorial Team,

Thank you very much for taking the time to review our manuscript “Optimisation strategies for directed evolution without sequencing”. We appreciate the thought and feedback provided by all reviewers, in response to which we have significantly updated and improved our manuscript.. Please find below a list of responses to each point in the reviewers’ comments, containing descriptions of the changes made. Alongside this letter we enclose an updated version of the manuscript with changes highlighted, as well as a clean version of the new manuscript.

Reviewer #1: *In this paper, the authors present several approaches for improving the efficiency and performance of directed evolution experiments, without a need for detailed sequencing data. This is achieved through the implementation of a threshold for selection and a “base chance” parameter that enables exploration and exploitation to be tuned, and the inclusion of population splitting to enable the exploration of multiple different paths that a population can take through the fitness landscape. Using theoretical and empirical fitness landscapes they demonstrate that their approach outperforms standard approaches.*

Overall, I found this to be an enjoyable and mostly well-written paper that tackles an increasingly important challenge in bioengineering. The work is perfectly aligned to the readership of PLOS Computational Biology, and I believe will be an important contribution to the field. Specifically, the ability for these relatively simple additions to greatly improve the chance of finding optimal solution during a directed evolution experiment make this work of great value and ensure its broad appeal to both theoretical and experimental audiences. I found the presentation of the results to be very clear and had no issue following the models and selection algorithms presented. I did have some more substantial comments (see below) on the scale of the theoretical simulations and access to code, but I believe these would require only a minor revision to address.

We appreciate the reviewer’s enthusiasm for our work approach, its contribution, and alignment with the PLoS Computational Biology readership. Below we go through the reviewer’s suggested changes and address them point by point.

It was unclear to me why N = 25 and K = 5 was used throughout to assess the selection scheme. It seems likely that features like population splitting, will play an important role in landscapes that are not too rugged or smooth. Better understanding that transition would be interesting to explore.

This is a very important point, and one that we discussed frequently over the course of the work. Initially we considered performing simulations across many different landscapes, and displaying the average of the results. However, doing so would require a major assumption about the expected distribution of landscape properties that would be encountered in experiments - which we do not know for more than a small number of examples *a priori*. In order to avoid making such an assumption we instead investigated the properties of a range of landscapes (e.g. varying N and K), and chose *one* set of parameters that was moderate in its behaviour, i.e. not possessing exceptionally smooth nor rugged characteristics. This, of course, is itself an assumption, but we believe more justifiable considering our work focuses on designing selection strategies suitable for variable/unknown landscapes (i.e. our work does not aim to predict/model the properties of landscapes across different natural systems). Nevertheless, we have made the addition of Supplementary Figure 1 to the work, which displays the behaviour of nine different NK landscapes (including our chosen landscape N=25 K=5) highlighting the generalisability of our results. We further observe that landscape structure does indeed play a role in population splitting performance (being more beneficial on rugged landscapes), and we have included a new Supplementary Figure 3 to display this trend.

I found the population splitting aspect of the work very interesting and wondered whether there was a simple mathematical relationship that could be used to calculate the optimal number of

sub-populations from the population size, N and K values. The data in Fig 3D looks smooth and so there might be quite a simple form that could be established. This would be helpful in making good choices, especially as N and K can be estimated from experimental data as the processes of evolution takes place.

In terms of the optimal number of sub-populations, we found that if we have n random variables with identical distribution, $X_1, X_2 \dots X_n$, (which are the outcomes of a directed evolution experiment with n sub-populations) then the max distribution is easy to characterise. $P(\max(X_1, X_2 \dots X_N) < x) = P(X_1 < x)^n$. So optimal population splitting (assuming populations of equal size) can be determined by characterising the distribution of outcomes across a range of population sizes, for the particular landscape in question. We have attempted, but not succeeded, in finding a mathematical relationship that integrates N and K from first principles - but this would certainly be interesting and will form a basis for future work that would explore this from a theoretical standpoint, alongside the influences of mutation rate and base chance.

On a broader point, while the above theoretical results would certainly be interesting, we do not believe they would not be a silver-bullet to design optimal strategies prior to any experimental work. As the reviewer suggests this would require ability to estimate N and K prior to or during an experiment. We do not believe that effective techniques yet exist for this challenge, and indeed the latter (estimating N,K from online experimental data) is one that we are currently also working on. This work will in the future build on the current paper, for example by informing how strategies could be dynamically updated during an experiment as more information becomes available.

I could not work out how to run the models in the linked code repository. It may be helpful to have a notebook that covers all the key types of simulation performed in the work to enable reproduction of the results and use by others.

Thank you for taking the time to run the code, we would like for it to be as accessible as possible. In response we have now restructured and streamlined our code at https://github.com/nesou2/directed-evo in order to make it more user-friendly. There are now only five base functions needed to perform a directed evolution simulation, and these can be found in direvo_functions.py. Also included is a tutorial in “examples.noteboook.ipynb” which walks the user through key examples of NK landscape and GB1 landscape runs.

“biased toward user-defined desirable variants” -> “toward desirable user-defined traits

Proposed change accepted.

“desirable output values” -> “desirable output expression levels”

This is indeed clearer, we have made the change.

Perhaps introduce the term “screening” that is typically used in the context discussed?

We have modified the introduction to use language related to “screening” throughout.

“increasingly high-dimensional information” – I agree that the data has a higher dimension (i.e., includes time), but not that the dimension is increasing beyond that. Clarifying this would be helpful.

Agreed, all the extra data does come from time so we could be more specific. To clarify this, we have changed the phrase to ‘This produces large amounts of additional temporal information’.

Does the trait-of-interest undergo directed evolution? Is it not the biological entity?

Changed to ‘biological entity undergoes directed evolution’.

“variants with each iteration” -> “variants during each iteration”

Proposed change accepted.

“which in particular enable effective” -> “which enable effective”

Proposed change accepted.

“e.g. FACS” -> “e.g. via FACS”

Proposed change accepted.

“of the distribution Fig. 2C” -> “of the distribution. Fig. 2C”

Fixed - thank you for pointing this out.

“dataset to to reduce” -> “dataset to reduce”

Fixed.

It may be useful to have a short paragraph in the introduction giving some background on emerging in vivo mutagenesis methods.

Given that *in vivo* mutagenesis methods are such an important part of the directed evolution pipeline in this work, this is certainly something we should have included previously. We have updated the introduction with an additional paragraph describing what *in vivo* mutagenesis means, and providing references to emerging *in vivo* mutagenesis methods.

Figure 2: For the normalised fitness measures, it would be helpful to see the spread that is associated with the average presented. I assume it would be feasible to include the standard deviation in these values? I’m wondering how this is affected by the parameters.

We have now included standard deviation in our normalised fitness measures for Figure 2 and also Figure 4, and we think this is a welcome addition. Note that these measures of spread represent the variation between trials when a larger number are run, *rather* than (for example) uncertainty in the mean value estimated over those trials. Due to the significant amount of randomness in the process the bars are overlapping, but we don’t believe that this undermines the trends that we describe.

All Figures: While the content of the figures is excellent and well-presented, the text labels in most of them was often too small to read comfortably. I would suggest considering increasing the size of the text in all of them to ensure it is 8pt when present in the paper format and possibly carrying out minor reorganisation/resizing of the panels where this is challenging.

To improve the readability of our figures, we have performed necessary adjustments, including font size and resolution increases.

Reviewer #2

In this work, James and collaborators present a thoughtful discussion of strategies for optimizing directed evolution experiments that do not rely on sequencing. Using a variety of simulations, they show that tweaking different features of the directed evolution process, including the mutation rate, method for selection, and number of populations, can be advantageous in different scenarios. This is a good point, which experimentalists should take to heart.

While the concept presented here is interesting, it's a bit more difficult to connect that concept with practice. The authors present some work based on real data, but I have questions about this process as well. My major comments are below.

We are thankful for the reviewer's positivity about our work; we address each of their comments below point by point.

Application in practice. The authors describe a number of different parameters that can be tuned to affect directed evolution experiments, including varying mutation rates, selection functions, and so forth. However, which choices are "better" and which ones are "worse" depend on the underlying fitness landscape. For example, when using a step function for selection, increasing the base chance of selection is helpful for rugged landscapes but harmful for smooth ones. In Figure 3, the outcome of the simulated experiments is a nonlinear function of the number of subpopulations, with the optimal performance found somewhere between the maximum and minimum numbers. Beyond knowing that these variables are ones that could, in principle, be experimentally manipulated, how would the results described here be used in practice?

This is a very important point, and one that we discussed at length amongst ourselves. The applicability of this work can be considered in two contexts. First, we found various broad conclusions regarding improvements that could be made to experiments (i.e. including a small amount of population splitting) which appear to be beneficial *independently of* population size in realistic population size settings, and thus could be used *in general* by experimentalists. Beyond this, there are other clear improvements that can be made (e.g. with the optimised base chance), but which are not generally true in varying contexts (e.g. varying N,K). Nevertheless these approaches would become applicable in practice if supplemented by methods for landscape inference, which fortunately is an increasingly active field (and one that we ourselves have recently contributed to in another publication). Following this discussion, we have added an additional paragraph to the discussion to address this comment, which outlines several landscape inference approaches that could act to support this work in future. We believe this work would go hand in hand with landscape inference methods, to maximise their potential in directed evolution.

Shape of the fitness landscape. Several different landscapes are used in this work, including NK landscapes with different parameters and an empirical landscape derived from ref. 19. The best approach to optimize directed evolution depends upon the shape of the landscape. Are there typical landscapes that experimentalists are most likely to encounter? If so, what evidence supports this? How would one know the shape of the landscape, and thus what directed evolution approach to use, in a novel experiment?

Building on our response to the previous point, as our ability to measure large-scale empirical landscapes improves, we will be able to answer this question with increasing accuracy. The main body of our work leveraged the highest-quality example of an empirical fitness landscape that was available (ref 19). Since we originally submitted our work to PLOS Computational Biology, an additional landscape of equal size was published, and we have been able to run tests on this data set and add results to our paper as part of the extended Figure 5. This landscape demonstrates similar

properties as the previous one - despite measuring a significantly different protein fitness in different biological context - which supports our suggestion that the standard approach to directed evolution (without any splitting/base chance), is sub-optimal, and that a small degree of base-chance and/or population splitting may improve outcomes in practice. As the bank of large fitness landscapes grows, we will certainly be highly interested in answering the question of a “typical” landscape. Similarly, we (and others) are continuing to develop methods for real-time estimation of landscape properties from experimental data, which as described above could allow optimisation of the designed selection strategies in real time.

To coalesce some of the above points we have updated the new paragraph in the discussion to describe how using fitness landscape inference techniques it will still be possible to obtain some information about the potential shape of a fitness landscape in a novel experiment, prior to starting.

Fitness in simulations. It appears that the authors assume that some metric of fitness can be measured without noise in realistic data. This seems unlikely. How would these methods work if precise fitness measurements were not available? For example, one could consider some measurable quantity (e.g., fluorescence intensity) to be a noisy measurement of a true, underlying component of fitness.

It is certainly true that noise in fitness measurements will be a key challenge in this framework, and also that the noise in a given fitness measurement will be highly context/experiment dependent. A benefit of our approach - which selects based on a variant’s *ranking* in a population rather than *absolute* fitness - is that it is robust to systematic and experimental errors such as uniform scaling factors or offsets in measurements which often lead to experiment-to-experiment or instrument-to-instrument variability in bioscience experiments. Nevertheless, measurement *noise* could, as the reviewer states, lead to a re-ordering in this ranking and hence perturb our selection methods. To explore how robust the strategies are to noise, we have produced some additional simulations (Supplementary Figure 2) showing the impact of noise on base chance performance (which we believe is more likely to be impacted by noise than the splitting performance).

We find, as expected, that the method can withstand a small amount of noise, although significantly greater than 5% noise (in terms of ordering of a population ranking) can reduce performance to the level of the no-base-chance case. However, an interesting conclusion can still be drawn from this, which is that noise *itself* may be playing a similar role to base chance, by promoting the inclusion of less fit variants.

Application to real data. While the simulation using an empirical landscape suggests that alternative experimental approaches could improve the results of directed evolution, this is far from a proof in practice. When the shape of the fitness landscape is known, it is not surprising that it is possible to generate post hoc an experimental process that more reliably approaches the fitness maximum. For a novel data set this would be challenging. As discussed above, different experimental choices can either help or impede evolution, depending on the (unknown) shape of the underlying landscape. Without an application, it is difficult to appreciate the utility of this approach.

Following the reviewer’s suggestion we have added to the new paragraph about the challenge on landscape inference in the discussion section of our manuscript. In particular, we and others are working on methods so that in future it may be possible to estimate the ruggedness of a protein fitness landscape from data available prior to and during an experiment. Landscape ruggedness is broadly a result of interactions between residues in a protein, and such information could be gathered from multiple sequence alignments between species (which provide information about co-evolution), or protein sequence-structure models. We have also added reference to recent papers that explore ruggedness analysis for experimental datasets such as DOI: 10.1126/science.adh3860, as well as

other recent works released after our manuscript was submitted which investigate the impact of selection stringency in simulation (DOI: 10.1101/2024.06.09.598029). Together with our work we believe these publications highlight how in future we may be able to put such analyses upstream of our directed evolution strategies to inform the optimal approach.

Figure 2 C and D are reversed in the caption.

Resolved - thank you for pointing this out.

The term in vivo mutagenesis is a bit confusing. After reading the linked review, I understand: this refers to mutation within a replicating cell, rather than artificial mutagenesis to generate phenotypic variation. But the directed evolution experiment itself is certainly in vitro. It may be helpful to add a sentence that explains this concept more precisely.

To add some clarity about the definition of *in vivo* mutagenesis (and as also requested by Reviewer #1), we have included an additional paragraph to the introduction describing what this phrase means, and providing references to primers on *in vivo* mutagenesis methods.

We would like to again thank both reviewers for their comments on all aspects of our paper; having considered and addressed these we believe the updated submission is now a much improved paper.
