# Supplementary material for: Optimisation strategies for directed evolution without sequencing
Source: PLoS Comput Biol. 2024 Dec 19;20(12):e1012695. doi: 10.1371/journal.pcbi.1012695 (PMC11698521; doi:10.1371/journal.pcbi.1012695)
Supplement: Attachment — Submitted filename: Reviewer_Comment_Reponses_Round2_HS 1.pdf [file pcbi.1012695.s007.pdf]

To the Editorial Team,

Thank you very much for taking time to assess our response and provide further suggestions. We are grateful to have received further feedback following the first round of review, and in response to this have conducted a series of new simulations to address suggestions from Reviewer #2. These have been integrated into a new supplementary figure, as well as updates to the results/discussion throughout the text. Please find descriptions of our responses and modifications below.

Reviewer #1: *I commend the authors on fully considering and addressing my concerns and strengthening this work. I am happy to now support for publication.*

Thank you very much for supporting the work, and providing such valuable feedback in the previous round. We are glad that the updates to the work met your satisfaction.

Reviewer #2: *While the authors have responded to prior comments, significant doubts remain. The most essential point is how the methods the authors recommend could be applied in practice when the shape of the fitness landscape is unknown.*

There are several comments made in the response to reviewers which did not appear to make it into the paper, but in the Discussion the authors state that "the continued development of fitness landscape inference tools, however, we may be able to estimate such parameters in advance (or during) a directed evolution experiment." At the level of discussion in the paper, this seems circular: if the landscape is already known by other means, then what is the purpose of the experiment? Can this point be clarified?

In this paper we mention two key categories of landscape inference tools: Those that can be done using pre-existing data (i.e. multiple sequence alignments, refs 51, 52, 54) and those that are conducted during an experiment (refs 39 and 55). To clarify this distinction we have re-written paragraph 3 of the Discussion to clearly define these two categories, as well as how our work fits with recent/ongoing work on inference of fitness landscapes from real-time experimental data.

The first category of landscapes, those based on pre-existing sequencing, have the key limitation of requiring information (i.e. multiple sequence alignments) that is likely not available when addressing novel engineering challenges (hence motivating our study on the whole – trying to optimise directed evolution methods for when such prior data is not available). The second category of approaches are landscape inference tools which can be implemented *during* an experiment. For example, reference 55 outlines such an approach (which we now highlight towards the end of Discussion paragraph 3) whereby a NK-trained neural network is used to make ruggedness predictions from the overall behaviour of a randomly mutating population. Instead of producing specific predictions

of peaks on the landscape, this phenotypically driven tool produces "global" (or at least local) measurements of ruggedness. We believe this combination to be particularly interesting, as it results in an entire pipeline with no sequencing at all.

We would also like to clarify that this class of methods in particular does *not* lead to a situation of circularity i.e. if the landscape inference tool is used the experimentalist could just choose the highest peak. This is because such methods are only estimating *parameters* of landscapes (e.g. statistical measures of ruggedness) rather than measuring the landscape directly (i.e. a gene sequence to function mapping). Thus, while they allow you to learn parameters of the landscape in real time, all these parameters can do is update your experimental strategy to optimise the chance of finding a highly performing variant *rather than identifying that variant directly*.

As such, our overall conclusion (supported by our theoretical data and testing on experimental datasets discussed) is that in these cases (which are further motivated in the manuscript – see discussion of points below) by use of tools that inform both strategy selection (i.e. base chance and splitting), as well as promising starting points for directed evolution, one can achieve a superior overall pipeline.

We have updated paragraph 3 of the Discussion, to clarify this point and address the interpretation of a circular argument.

More generally, the authors should precisely articulate how their ideas should be used, as well as the evidence for them. If, as stated in the response, there are some approaches that should be useful in general (regardless of the shape of the landscape), then the authors should say this clearly and support this claim with evidence. Of course, this does not imply that methods must be tested against all possible landscapes, which might as well be infinite in number. Simulations using a variety of easily parameterized landscapes would be sufficient to argue for the general use of a method.

In order to explain to the reader how these methods could be implemented, we have produced a new schematic (Fig. 5E), which outlines an example pipeline. It suggests two options: Either the experimentalist performs some landscape inference, and uses this to inform their strategy, or they employ the default NK strategy. The two different approaches from the schematic are discussed in the newly added final two paragraphs of Results.

To produce the "default NK strategy", we have taken on the suggestion of Reviewer #2 and conducted a wide parameter sweep over a variety of landscapes and experimental conditions (population size, mutation rate). The outcome is presented in Fig. S4, including both the mean performance of all strategies over the various conditions, as well as a side-by-side comparison of the default NK strategy to the current standard

approach (no base chance, no splitting), in histogram format. This is discussed in the penultimate paragraph of the Results.

In terms of how someone can physically implement our methods, we have also added a new paragraph to the Discussion (paragraph 2), which explains in more detail how these methods are employed experimentally under different conditions.

We would like to note that one thing we have not done is implement the landscape inference tools ourselves as part of our pipeline. Although we very much believe this is possible, and it is something we intend do in the future, it remains a significant challenge. The process of closing this gap involves either finding a reliable way to investigate the relationship between real landscapes and NK landscapes, or performing many rounds of directed evolution experiments to evaluate how the output of landscape inference tools corresponds to the performance of different strategies via experimentation. This is discussed both in the final paragraph of Results and the third paragraph of Discussion. We leave this challenge open to further work.

If, on the other hand, the authors do not believe (or do not have evidence to support) the general applicability of different methods, then this should also be discussed clearly in the paper.

In general, we have made the decision to soften the language of the paper (most notably in the Abstract and Author Summary). Because we agree that we cannot guarantee that experimentalists will be able to select a *guaranteed* best strategy using our method at present. Therefore, we have adapted the focus of these sections to be more on the *sub-optimality* of typical default strategies. Please refer to the highlighted changes in the Abstract, Author Summary, Introduction, and final paragraph of Discussion. For instance, "can be implemented" -> "could in future be implemented", and sentences such as "There are, however, many possible approaches to exploring non-convex evolutionary fitness landscapes, and choosing [the no base chance, no splitting] strategy as default may not always be the strongest approach".

Alternately, if the idea is that experimentalists should learn something about the landscape before implementing some of the approaches described here – which seems to be the argument presented in the Discussion in the current version of the paper – then the authors should elaborate on this point more explicitly. How would they envision practitioners applying the population splitting/base chance ideas in a new experiment?

We hope that the schematic in Fig. 5E clarifies how we envisage this work to be implemented in a directed evolution pipeline. The two possibilities we suggest (either a default NK strategy from an NK parameter sweep, or a landscape inference-informed approach) are discussed in the newly-added final two paragraphs of the results respectively. We go into more detail on the availability of landscape inference options

available in Discussion paragraph 3. In terms of implementing base chance and population splitting in practice, we have written a new paragraph (Discussion paragraph 2) to discuss this explicitly. We have also made modifications to the opening and closing parts of the manuscript, offering clearer summaries from the outset about how this work should be imagined and used. All the new modifications are highlighted in the attached manuscript, and we hope they provide a clearer picture of our intentions overall.
